# Electron quantum path control in high harmonic generation via chirp variation of strong laser pulses

**DOI:** 10.1038/s41598-021-03424-3

**Published:** 2021-12-13

**Authors:** S. Petrakis, M. Bakarezos, M. Tatarakis, E. P. Benis, N. A. Papadogiannis

**Affiliations:** 1 Department of Physics, University of Ioannina, 45110 Ioannina, Greece; 2 Institute of Plasma Physics and Lasers, Hellenic Mediterranean University Research Centre, 74100 Rethymno, Greece; 3 Department of Music Technology and Acoustics, Hellenic Mediterranean University, 74133 Rethymno, Greece; 4 Department of Electronic Engineering, Hellenic Mediterranean University, 73133 Chania, Greece

**Keywords:** High-harmonic generation, Nonlinear optics

## Abstract

The quantum phases of the electron paths driven by an ultrafast laser in high harmonic generation in an atomic gas depends linearly on the instantaneous cycle-averaged laser intensity. Using high laser intensities, a complete single ionisation of the atomic gas may occur before the laser pulse peak. Therefore, high harmonic generation could be localised only in a temporal window at the leading edge of laser pulse envelope. Varying the laser frequency chirp of an intense ultrafast laser pulse, the centre, and the width of the temporal window, that the high harmonic generation phenomenon occurs, could be controlled with high accuracy. This way, both the duration and the phase of the electron trajectories, that generate efficiently high harmonics, is fully controlled. A method of spectral control and selection of the high harmonic extreme ultraviolet light from distinct quantum paths is experimentally demonstrated. Furthermore, a phenomenological numerical model enlightens the physical processes that take place. This novel approach of the electron quantum path selection via laser chirp is a simple and versatile way of controlling the time-spectral characteristics of the coherent extreme ultraviolet light with applications in the fields of attosecond pulses and soft x-ray nano-imaging.

## Introduction

The rapid evolution in science and technology of femtosecond laser pulses during the last three decades has led to the development of intense and coherent electromagnetic fields (EM)^1^. Laser pulse peak intensities of the orders of $10^{13}{-}10^{16}$ W/cm$^2$ are routinely available in laboratories worldwide. The interaction of such strong laser pulses with atoms unveiled new strong field phenomena such as above threshold ionisation^2,3^, tunnelling ionisation^4^, over the barrier ionisation^5,6^, as well as high harmonic generation (HHG)^7^ allowing for coherent spectral sources in the XUV region. In such intense EM fields, the interaction between the laser field and the atom is governed primarily by the laser pulse characteristics (peak intensity, temporal frequency and phase) as well as the depth of the atomic Coulombic well. Consequently, semiclassical theories have been developed to describe such interactions, the most renowned being the three-step model for the HHG process^7,8^.

In the three-step model picture, the electrons are initially ionised via tunnelling or even over the barrier ionisation. Then, they are driven by the strong alternating laser electric field back and forth the atomic nuclei. There is a non-zero probability for the oscillating electron to recombine with the parent nucleus emitting the excess energy as high frequency radiation. The majority of the electrons, however, are emitted into the continuum resulting in a partially ionised medium. The above recollision process is repeated every half-cycle of the laser electric field as long as the medium is not fully ionised. This periodicity is manifested in the frequency spectrum of the emitted photons as a comb of odd order harmonics. Specifically, as the harmonic order increases, there is initially a region in the frequency spectrum where the harmonics signal exhibits a decrease (lower order harmonics), followed by an extended region of harmonics with approximately the same efficiency (plateau), and finally a region where the harmonics signal rapidly decreases (cut-off).

**Figure 1:**
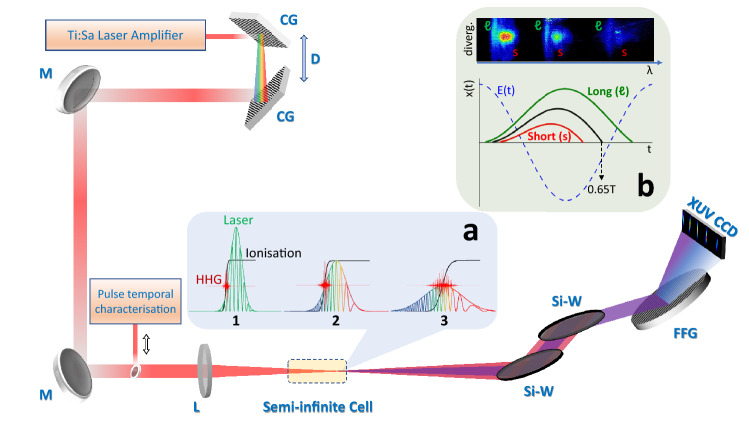
Schematic layout of HHG experiments performed with femtosecond high intensity chirped IR Ti:Sa laser pulses. Femtosecond amplified laser pulses are chirped by varying the spacing between the compressor gratings (CG). Laser high harmonics are generated after focusing the chirped laser pulses on a semi-infinite noble gas (Ar) cell. High harmonic spectra are measured by an XUV CCD camera after IR filtering by silicon wafers (Si-W) and XUV dispersion by a flat-field XUV concave grating (FFG). (**a**) HHG E-fields for three cases of chirped laser pulses having the same energy; (**a1**) Fourier limited (unchirped) pulses; (**a2**) Low negatively chirped pulses; (**a3**) High negatively chirped pulses. Note that HHG occurs at the same laser pulse intensity (controlled by electron ionisation) corresponding to different time instants of the laser intensity envelope. (**b**) [Bottom] Schematic of electron trajectories within an E-field laser period leading to the recollision with the parent nucleus. [Top] HHG spectral images from long and short electron trajectories exhibiting different spectral and geometrical characteristics. D: Adjustable grating distance; M: Low dispersion fs mirror; L: Low dispersion thin focusing lens.

There is an infinite number of quantum paths that the recolliding electron can follow. However, for the same electron return energy, there are only two surviving paths that result in a recollision with the parent ion during the first laser cycle. For these two paths, both ionisation and recombination time instants are different. One of the paths lasts shorter time than the other and the paths are consequently termed *short* and *long* trajectories, respectively. Specifically, the short trajectory duration is shorter than 0.65*T*, where *T* is the laser pulse period, while the long trajectory duration is longer than 0.65*T*, as shown in the inset (b) of Fig. 1. The quasi-classical action of the electron that determines the phase of the trajectory^7^ depends on the time spent in the continuum while it also depends linearly on the intensity of the driving laser pulse^9^. The total quantum phase of the *q*th-order harmonic is $\phi (q) = a_s I(t) + a_l I(t)$, where *I*(*t*) is the cycle-averaged laser pulse time-dependent intensity, while the parameters $a_s$ and $a_l$, with $a_s \le a_l$, correspond to the short and long trajectories, respectively, and depend on the duration of the quantum path. The *q*th-order harmonic signal results from the coherent summation of the aforementioned quantum paths and corresponds to an energy of $E = q \hbar \omega _L$, where $\omega _L$ is the laser central frequency.

The multi-cycle temporal characteristics of the plateau harmonics depend strongly on the phase difference between the long and short trajectories which is time-dependent, since the temporal intensity varies for every laser field cycle. Moreover, for the plateau harmonics, the time duration of the short and long quantum paths is markedly different, thus $a_s<< a_l$. As the cut-off region is approached, the duration of the paths becomes comparable, and at the cut-off the two quantum paths become degenerate (intermediate trajectories) with $a_s \simeq a_l$. Thus, cut-off harmonics are favourable in coherent summation applications. Indeed, the initial attempts for generation of attosecond beatings, utilising coherent summation of harmonic combs, were largely oriented in the cut-off region^10–12^. However, a significant obstacle in generalising these attempts was that the cut-off harmonics inherently suffer from low efficiency.

It becomes evident from the above discussion that controlling the relevant contribution of long and short trajectories of the plateau harmonics, is of fundamental significance, not only for the detailed understanding of these phenomena, but also for applications such as attosecond pulses generation^13,14^ and coherent diffraction imaging^15–18^, where the geometrical and phase characteristics of the harmonics beam play a major role. The main distinctive footprint of the two trajectories in each plateau harmonic is the difference in their divergence. The long trajectory has a larger divergence than the short, due to the fact that the electron travels longer distances in the partially ionised medium during the recollision process^19^. In addition, for laser intensities where the ionisation of the medium is relatively high, the central wavelength of the two trajectories is also slightly different. This phenomenon is attributed to the different time dependence of the two trajectory phases, which leads to coherent summation for each trajectory at different wavelengths (double-peaking phenomenon)^20^.

The origin of divergence of the two paths in HHG has been investigated in a number of early publications^7,9,21–23^, while attempts to separate and selectively control the two trajectories have also been reported. Bellini et al. were the first to experimentally demonstrate the different divergence distribution of the two trajectories using interferometric methods^24^. Their results were soon adopted by the attoscience community to filter the long trajectory and achieve high quality attosecond pulse trains^14^. Willner et al. used a multi-jet arrangement of two gases showing a clear spectral separation of the two trajectories^25,26^. Ishii et al. used a phase-locked two-colour ($\omega -2\omega$) laser field to demonstrate the control of the electron trajectories near the cut-off region, through the relative phase between fundamental and second-harmonic fields^27^. Brugnera et al. varied the phase of a two colour driving field and demonstrated near complete control over the long trajectory, which was easily separated spatially from the short^28^. Recently, Abbing et al. used tailored laser pulses consisting of the fundamental and its intense orthogonally polarised second harmonic in a two-colour HHG scheme^29^. By controlling the relative delay between the two fields they showed the suppression and enhancement of long and short electron trajectories, respectively. The authors emphasised on the necessity to develop robust methods to control the divergence of the generated EUV beam, via the trajectory selection, as an indispensable tool for improved imaging techniques based on coherent diffraction imaging.

In this article we present a novel method for the coherent control of the two quantum paths in plateau harmonics generated in a noble gas, based on the temporal rearrangement of the spectral content of the high intensity generating ultrashort laser pulses, namely the *chirp* of the laser pulse. This control over the time-dependent frequency of the high intensity laser pulses, allows for the accurate control of the time-dependent slope of the cycle-averaged laser intensity, the temporal evolution of the ionisation degree of the medium within the laser pulse, and the self-phase modulation of the laser pulse inside the partially ionised medium. Since the temporal phase of each trajectory depends on all the above factors, which are all simultaneously controlled by our method, we were able not only to resolve the two trajectories but also to accurately control the efficiency with which these are generated. Specifically, we found experimental conditions under which the only surviving trajectory was the short one. We have also developed a phenomenological model, which takes into account the aforementioned physical processes, that supports our findings. The conceptional background and experimental realisation of the method are illustrated in Fig. 1.

## Results

### Electron trajectory control

**Figure 2:**
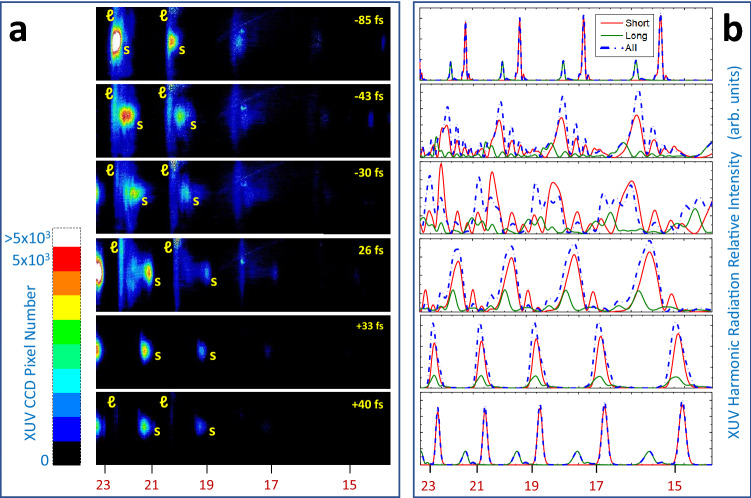
(**a**) Representative measurements of XUV harmonic spectral images for various laser pulse durations controlled by the imposed chirp. The harmonics spectral locations, estimated according to the experimental setup optical geometry, are noted at the bottom of the figure. Negative and positive signs of the laser pulses durations correspond to negative and positive laser pulse chirp, respectively. The spectral separation between the short and long trajectories, indicated by the letters “$\ell$” and “*s*” in the spectral images, respectively, is clearly demonstrated for certain chirp values. Note that for the duration of +33 fs, the long trajectories contribution to the HHG is severely suppressed, thus leaving only the HHG by short trajectories in the spectral image. (**b**) XUV harmonic spectra obtained with our model calculations corresponding to the experimental conditions of their counterpart XUV harmonic spectral images in (**a**). Both short and long trajectory contributions, as well as their coherent addition, are presented. It is seen that the calculations fairly reproduce the main qualitative characteristics of the measured spectral images. Note that the 17th and 15th order harmonics are seen to be strongly suppressed in the experimental spectral images due to their strong absorption by the Ar gas, a condition not included in the simulations.

Using the experimental setup described in “Methods” section, we were able to accurately control the laser pulse timedependent intensity and frequency through the laser pulse chirp variation. We have performed a systematic investigation of HHG in argon gas semi-infinite cell measuring the high harmonic spectrum as a function of the generating laser pulse chirp. Typical plateau harmonic spectra generated in 60 Torr Ar filled semi-infinite cell for various generating laser pulse chirp values are presented in Fig. 2a. All the measurements were performed maintaining a constant laser pulse energy at 1.0 mJ as well as the same focusing conditions, i.e., *f*-number = 4 and focus at the exit pinhole ($\sim 100 \, \upmu \text {m}$ in diameter) of the semi-infinite cell. The laser peak intensity for the Fourier transform limited (FTL) pulse of 26 fs (the shortest possible) was experimentally assessed to be $2.3 \pm 0.5 \times 10^{15} \, {\text {W/cm}}^2$. The pulse durations indicated in the spectral images in Fig. 2a were measured via an intensity background-free autocorrelator, while the plus and minus signs correspond to the positive (frequency increasing with time) and negative (frequency decreasing with time) chirp values, respectively.

As it is clearly seen in Fig. 2a, the harmonics spectral locations are blue shifted with respect to their positions estimated according to the setup optical geometry. This is a known behaviour in the literature^20,30,31^ and it has been identified as the result of mainly two mechanisms^32^. The first mechanism is the propagation effects of the laser pulse in the ionised medium. The partial ionisation of the generating medium during the laser pulse induces a variation of the refractive index^33^ that leads to a blue shift of the generating laser field^34^ and thus to a blue shift of the generated harmonics. The second mechanism is the nonadiabatic response of the electronic dipole to the rapid change of laser fields. Since the laser intensity is rising in the leading edge of the pulse, the recolliding electrons gain more energy with each successive laser field cycle. Therefore, the HHG at the leading edge of the laser pulse, conditions met in our study, induces a positive-chirp role in HHG, thus leading to a blue shift in HHG^35^. In addition, the 17th and 15th low order harmonics are seen to be strongly suppressed in the spectral images, as a result of their strong absorption by the Ar gas^36^.

In accordance with the above interpretations, it is seen that in our spectral images the harmonics generated by negatively chirped laser pulses exhibit an overall stronger blue shift that increases with increasing the negatively chirped laser pulse duration. However, for certain chirp values, the blue shift is not the same for the short and long trajectories, as evident for the cases of laser pulse duration-43 fs and -30 fs, as well as the FTL case of 26 fs. For these cases, the long trajectories, identified by their larger divergence, exhibit a stronger blue shifting, most possibly due to the delicate interplay between the above-mentioned mechanisms and the fact that the long trajectory travels longer distances in the partially ionised medium. This delicate interplay that results in the clear separation of the long and short trajectory is effortlessly realised in our method by simply varying the laser pulse chirp values in high intensity laser pulses that promote HHG at the leading edge of the pulse.

The most significant finding of our study, however, is that at a certain laser pulse chirp value, which corresponds to the laser pulse duration of +33 fs, the HHG by long trajectories is severely suppressed, thus leaving only the HHG by short trajectories in the spectral image. It is important to mention here that the experimentally observed suppression in Fig. 2a is fully within the dynamic range of our 16-bit X-ray camera and it is not a matter of color scale choice. This observation is of exceptional importance not only for fundamental Physics but also for applications in coherent diffraction imaging as well as in attosecond science. Indeed, for high quality imaging applications the presence of both trajectories introduces chromatic aberrations^29,37^. For a qth-order harmonic, the different contributions of the short and the long trajectories result in different virtual generation positions. Thus, when the harmonic is refocused, the images of the long and short trajectories will not be fully overlapped at the focal area, thus leading to an overall smearing of the image profile. In addition, larger divergent beams reduce the numerical aperture of an optical system. Similarly, in attosecond science, the presence of both trajectories largely degrades the quality of the formation of attosecond pulse trains and their corresponding use. Therefore, the controlled suppression of the HHG long trajectory leaving out only the HHG short trajectory as the coherent source is of vital importance for high quality HHG. To the best of our knowledge this observation has not been reported in the literature so far for HHG in a single atomic gas. Thus, the understanding of the underlying physical processes is of fundamental importance for the control of this behaviour.

### High harmonic spectral calculations

Using Eq. (6), presented in “Methods” section, we simulated the experimental conditions and calculated the spectral intensity of the high harmonics in the region of interest. The aim of these model simulations was not to reproduce in detail the experimental HHG spectra but rather to qualitatively reproduce their main features and behaviour by using acceptable parameter values. For this purpose, we used the following values for the parameters of the model: (a) Laser pulse: (i) $\lambda = 807\,{\text {nm}}$; (ii) $\tau _{FTL} = 26\,{\text {fs}}$; (iii) Peak intensity for the FTL pulse $I_0(\tau _{FTL}) = 2.3 \times 10^{15}\,{\text {W/cm}}^2$. (b) Argon generation medium: (i) refractive index $n_0 = 1.0000$ for 60 Torr pressure; (ii) nonlinear index coefficient $n_2 = 1 \times 10^{-20}\,{\text {cm}}^2/{\text {W}}$ for 60 Torr pressure^38^; (iii) $z_p$ is the distance of the laser beam propagating in the plasma and was estimated from the plasma luminescence images of the generating medium taken during measurements. Typical values are in the range of $1{-}2.5 \, {\text {mm}}$ depending on the chirped pulse duration; (iv) $z_g$ is the propagation length of the laser beam in the Ar medium before the HHG region which we estimate as the Rayleigh length minus the plasma length experimentally measured for each laser chirp. The measured plasma luminescence varies along the k-vector of the laser (differently for each laser chirp value) being strongest at the area closest to the exit pinhole of the semi-infinite cell, where HHG is efficient. By integrating the plasma luminescence along the plasma channel, a single plasma luminescence value is extracted at each laser chirp. By corresponding the maximum luminescence value to the maximum electron plasma density that can be reached for a singly ionised 60 Torr Ar gas density ($1.5\times 10^{18}\,{\text {cm}}^{-3}$), an average electron plasma density, that the laser beam experiences along its propagation inside the plasma channel, is extracted. The temporal function of the electron plasma density at the HHG area, $\rho (t)$, see Eq. (5) in “Methods” section, is estimated by multiplying the previously extracted average plasma density with the temporal ADK ionisation probability, see Eq. (8) in “Methods” section. (c) HHG phase parameters for the short and long trajectories, respectively^19^: (i) $\alpha _l = 13\times 10^{-14}\,{\text {cm}}^2/{\text {W}}$; (ii) $\alpha _s = 0.5\times 10^{-14} \, {\text {cm}}^2/{\text {W}}$. Note that some parameters are experimentally determined while others are acceptable values from the literature. The HHG phase parameters $\alpha _l$ and $\alpha _s$ were kept at the same values for the five plateau harmonics of the simulated spectral region since this is an acceptable approximation for close neighbouring plateau harmonics. We emphasise that small variations in the above values did not seem to appreciably alter the final results, maintaining the constraint that $\alpha _l>> \alpha _s$, which is well established in the literature^19^. In addition, the high harmonic phase offset $\Phi _{s/l}^0$ was set to zero, since it only corresponds to an offset in the harmonic E-field interference of the short and long trajectories, a usual assumption in similar studies^19^. Finally, it is a common observation in our HHG spectra, for the conditions where the high harmonics generated by the short and long trajectories are spectrally separated, that short trajectory high harmonic exhibit a higher signal than the long trajectory high harmonic. We experimentally estimate an overall acceptable ratio of $C_l/C_s \simeq 0.6$, and thus this value was adopted in our simulations.

In Fig. 2b the spectral model calculations (Eq. 6) are presented for the conditions that simulate the corresponding experimental spectral images in Fig. 2a, as described above. It is seen that the calculations fairly reproduce the main qualitative characteristics of the measured spectral images as they predict: (a) The spectral separation of the HHG for the long and short trajectories at the corresponding experimental pulse durations; (b) The coherent mixing of the two quantum paths when not appreciably spectrally separated (e.g. for the 26 fs case); (c) An increase in the ratio between short and long trajectory contributions for the case of the optimal pulse chirp (+33 fs), as compared to the severe suppression of the long trajectory observed in the corresponding experimental data. We attribute this to the fact that our model treats the problem as single atom response. Note that in the spectral model the self-absorption of the high harmonic in argon medium was not included in the simulations. This explains the presence of high harmonic intensity in the spectral region of the 15th–17th harmonics, as opposed to the experimental spectral images.

**Figure 3:**
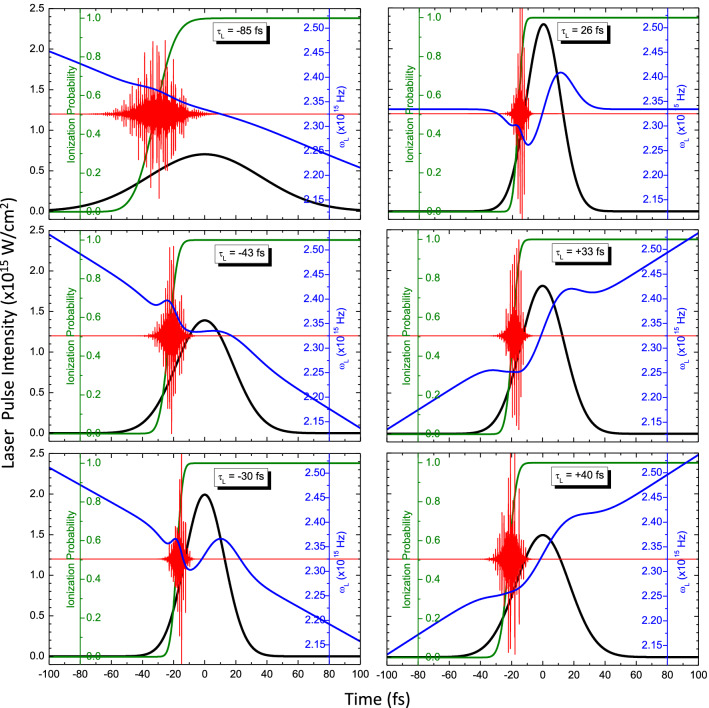
Details of our model calculations corresponding to the cases presented in Fig. 2. Black line: Laser intensity; Blue line: Laser angular frequency accounting for the linear laser chirp and SPM effects in the non-linear Ar medium and the plasma; Green line: ionisation probability of Ar according to the ADK model; Red line: Generated XUV radiation E-field, arbitrarily scaled along the y-axis in each graph for presentation purposes.

Additionally, in Fig. 3, the temporal variation of the plateau harmonics E-field, corresponding to the spectra of Fig. 2 (Right), is presented (red line, Eq. (1)). In the same figure, the laser intensity (black line, Eq. 2), the angular frequency (blue line, Eq. 3) and the medium ionisation probability (green line, Eq. 8) are also presented as a function of time in the same temporal window. The common feature in all of the graphs of Fig. 3 is that HHG takes place along the leading edge of the laser pulse at a variable with the laser pulse duration intensity interval, determined by the medium ionisation around its half-maximum value. For higher intensities, medium ionisation prevails and HHG is diminished. Note that, for the high laser pulse intensities, which correspond to small chirp values, the laser angular frequency is strongly modulated due to SPM effects. It should be emphasised that this strong modulation of the laser angular frequency around the FTL value of the laser pulse duration, along with the temporal variation of the laser intensity along the HHG intensity interval are fundamentally responsible for the structure of the spectral images, as seen from Eq. (1). Thus, SPM effects play a major role in the generation of high quality harmonics corresponding essentially to short trajectories.

## Discussion

Our experimental results and theoretical treatment clearly show that the frequency chirp of the ultrafast laser pulses is a crucial parameter for the control of the electron quantum paths in the HHG from atomic gases. As a consequence, this control affects not only the HHG spectra, by separating the footprint of the short and long trajectories in the plateau harmonics, but also significantly affects their relative generation efficiency. We clearly demonstrate in this work that for certain experimental conditions, easily achieved with nowadays HHG installations, the HHG efficiency of the long trajectory is dramatically reduced compared to that of the short trajectory, simply by adjusting the frequency chirp of the driving laser pulse to an appropriate value. This is attributed to the interplay of the inherent quantum phases for each quantum path, controlled by the fine adjustment of the HHG temporal window within the laser pulse envelope, the slope of the temporal intensity at this window, and the temporal frequency of the laser pulse. The above conditions are met for driving laser intensities strong enough to cause a significant degree of ionisation in the generating medium, that defines the temporal window for HHG. It should be noted here that for the laser pulse characteristics and varying the Ar gas pressure, the suppression of the HHG from the long trajectory routinely appears for small positive chirp values and corresponding pulse durations in the range of +30 to +40 fs. This is fully supported by our spectral model calculations since small variations in the values of the physical parameters of the model do not appreciably alter the findings.

Compared to previously reported methods^22,24–29^, the simplicity of our experimental method, that is based upon the appropriate selection of (a) the intensity of a single driving laser field, (b) the atomic target, and (c) the laser frequency chirp, easily controlled by detuning the ultrafast laser compressor gratings, signifies the applicability of our method as well as its potentiality for applications. In addition, since our method utilizes a semi-infinite cell, the generating medium can be easily kept at the same conditions. Thus, our method is attractive for the new generation high average power MHz amplified fs laser systems, which is the new trend for high averaged power XUV high harmonic sources, favourable for imaging applications and for basic research in storage ring accelerators.

We especially emphasise on our finding that, under certain experimental conditions, plateau harmonics are fully attributed to the short electron trajectory. The geometrical characteristics of such coherent harmonic radiation significantly favours coherent diffraction imaging applications in the scale of few-tenths of nanometers^29^. Furthermore, high efficiency plateau harmonics generated by only the short trajectory, achieved by our method, could be used in the attosecond pulse formation, since the produced attosecond pulses exhibit single sign frequency chirp, and thus could be compressed to their FTL value. Note that these attosecond pulses exhibit low divergence and thus achieve higher intensities with higher-quality foci favouring their use.

## Methods

### Experimental setup

The HGG experiments were performed using the experimental setup shown in Fig. 1. The generated higher order harmonics of the 26 fs, 1 mJ laser pulses (Amplitude Technologies), focused at the exit pinhole of an Ar gas filled static cell (semi-infinite cell), propagate along with the laser beam to a high vacuum chamber. The XUV light is filtered from the IR using a pair of Si wafers placed at the Brewster angle for the IR. The remaining of the XUV comb of high harmonics is diffracted by grazing-incidence flat-field diffraction grating (Hitachi 001-0639) allowing for high angular dispersion efficiency for the harmonics from 22 nm – 124 nm. The XUV light is detected at the focal plane of the harmonics of interest (15$\omega$–23$\omega$) using a high quantum efficiency 16-bit X-ray vacuum CCD camera (Raptor Photonics, Eagle XO) with a sensor having 2048 pixels (27.65 mm) at the grating diffraction axis and 512 pixels (6.90 mm) at the XUV divergence axis (perpendicular to the grading diffraction axis). The XUV grating and detector allow for a relatively high spectroscopic resolution from $\sim$80 to $\sim$100 pixels per 1 nm, on the CCD, for the spectral region shown. This resolution is an important experimental tool for the observations presented here. The value and the sign of the chirp of the laser pulses are controlled by tuning the compressor gratings of the laser amplifier relative to the position which delivers FTL laser pulses of 26 fs duration. For positive chirp values the lower frequencies appear at the leading edge of the laser pulse, while for negative chirp values the lower frequencies appear at the trailing edge of the laser pulse (see Fig. 1a). Assuming only linear chirp, its value is experimentally measured in shot-to-shot basis via a femtosecond single shot intensity autocorrelator (Amplitude Technologies, Bonsai) just before the interaction cell. The distance of the gratings in the laser compressor can be tuned according to the measured pulse durations and thus the laser pulse chirp is fully controlled during the experiments.

### Model Spectral Calculations

The developed spectral model for the high harmonics treats the problem as single atom response only temporally, omitting the spatial contributions like spatial phase matching, laser focusing, and self-phase modulation outside of the laser k-vector. It is an extension of the phenomenological model described by Carlstrom et al.^19^. The extension refers to the addition of the temporal dependence of the Ar gas ionisation and of the laser propagation in the generating medium that induces spectral variations owed to self-phase modulation (SPM) induced by the optical Kerr effect and by the plasma. This extension is necessary for our HHG conditions since the laser peak intensity is in most of the cases higher than the intensity inducing considerable Ar gas ionisation and thus the higher order harmonics are generated at the different temporal intervals of the leading edge of the laser pulse as seen in Fig. 3.

The electric field of the *q*th-order XUV harmonic radiation, corresponding to the short and long trajectories, symbolised with the subscripts *s* and *l*, respectively, can be written as^19^:

1 $$\begin{aligned} E^q_{s/l}(t)=C_{s/l}I^{n/2}(t)\exp \left[ iq\omega _{L}(t) t + i a_{s/l}I(t) + i\Phi _{s/l}^0 \right] , \end{aligned}$$

where

2 $$\begin{aligned} I(t)=I_0(\tau )\exp \left( -4ln2\frac{t^2}{\tau ^2} \right) \end{aligned}$$

is the Gaussian intensity temporal distribution for a laser pulse with FWHM duration $\tau$, $I_0(\tau$) is the peak intensity which depends on the laser pulse duration, and

3 $$\begin{aligned} \omega _{L}(t) = \omega _{L0} + \omega _{SPM}(t) + b(\tau ) t \end{aligned}$$

is the instantaneous laser frequency in the HHG region, including SPM modifications by Kerr-effect in the atoms and by the plasma, $\omega _{L0}$ is the central laser frequency and $b(\tau )$ the chirp parameter corresponding to the chirp added by the laser compressor and is given by

4 $$\begin{aligned} b(\tau )=\pm \frac{4ln2}{\tau ^2} \sqrt{\frac{\tau ^2}{\tau ^2_{FTL}} -1}, \end{aligned}$$

where $\tau _{FTL}$ is the FTL laser pulse duration. $C_{s/l}$ are weights for each of the short and long trajectories while *n* is a non-linearity parameter for the HHG conversion. $a_{s/l}$ are the dipole phase constants^23,39^ and $\Phi _{s/l}^0$ is a phase offset depending on the atomic properties. In our extended model, the time dependent laser angular frequency $\omega _{SPM}$ accounts for the SPM effects caused by the laser propagation in the non-linear medium and in the plasma, as^40,41^:

5 $$\begin{aligned} \omega _{SPM}(t)= -\frac{n_2 \omega _{L0}}{c} z_g \frac{dI(t)}{dt} + \frac{\omega _{L0}}{2 n_0 c \rho _c} z_p \frac{d\rho (t)}{dt}, \end{aligned}$$

where $z_g$ is the propagation length of the laser beam in the Kerr medium within the Rayleigh length, excluding the HHG region (i.e. plasma length) and $z_p$ is the plasma length. *c* is the speed of light, $n_0$ is the refractive index of the generating medium and $n_2$ the nonlinear index coefficient, which depends on the gas pressure and the atoms species. The total refractive index is expressed as $n(I) = n_0 + n_2 I(t)$. $\rho (t)$ is the density of free electrons in the plasma depending on the gas pressure and $\rho _c$ is the critical plasma density with $\rho _c = 1.7 \times 10^{21}\,{\text {cm}}^{-3}$ at $\sim$ 800 nm.

The HH spectral intensity is calculated as the absolute square of the Fourier transform of the coherent summation of the E-fields over the participating harmonics. For each harmonic the E-field of the short and long trajectory is considered. Thus, the spectral intensity equation reads:

6 $$\begin{aligned} I(\omega ) = \left| {\fancyscript {F}} \left\{ \sum _{q} [E^q_s(t) + E^q_l(t)] \right\} \right| ^2. \end{aligned}$$

In the model, we explicitly include the ionisation of the medium, which is of major importance for the HHG conditions, since it is the primary competing process for the laser intensities used in this work. We use the well known ADK model, appropriate for tunnelling ionisation that prevails for the laser intensity conditions used in the experiments. The generated electric field of the *q*th-order XUV harmonic radiation obtained in Eq. (6) is multiplied by the probability that the atoms of the generating medium have not been ionised by tunnelling ionisation, i.e.:

7 $$\begin{aligned} E^q_{s/l}(t)_{out} = [1-P(t)] E^q_{s/l}(t), \end{aligned}$$

where *P*(*t*) is the tunnelling ionisation probability up to the moment t, described as:

8 $$\begin{aligned} P(t) = 1 - \exp \left( -\int _{-\infty }^{t}W_{ADK}(t')dt' \right) . \end{aligned}$$

$W_{ADK}(t)$ is the time-dependent ionisation rate predicted by the ADK tunnelling theory, valid for our laser intensities, and it is described as^4^:

9 $$\begin{aligned} W_{ADK}(t)=|C_{l^* n^*}|^2 G_{lm} I_P \left( \frac{2 F_0}{F(t)} \right) ^{2 n^*-|m|-1} \exp \left( -\frac{2 F_0}{3 F(t)} \right) . \end{aligned}$$

where *l* is the orbital quantum number, *m* is the magnetic quantum number, $n^*$ is effective principal quantum number, $l^*=n^*-1$ is the effective orbital quantum number, $I_P$ is the ionisation potential of the atom, $F_0 = (2I_P)^{3/2}$, $F(t)[a.u.] = \sqrt{I(t)[{\text {W/cm}}^2] / (3.55\times 10^{16})}$ the time dependent laser field strength, while $C_{l^* n^*}$ and $G_{lm}$ are parameters depending on the quantum numbers. For the argon gas, in use here, the values of the parameters are as follows: $I_P = 15.76$ eV, $F_0=1.24665$ a.u., $l=1$, $m=0$, $n^*=0.92915$, $l^*=-0.07085$, $G_{lm}=3$ and $|C_{l^* n^*}|^2 = 4.11564$.
